# Editorial: Clinical Updates on Bariatric Surgery

**DOI:** 10.3390/jcm12030894

**Published:** 2023-01-23

**Authors:** Claudio Gambardella, Ludovico Docimo

**Affiliations:** Division of General, Oncologic, Mini-Invasive and Obesity Surgery, University of Study of Campania “Luigi Vanvitelli”, 80131 Naples, Italy

Dear Editor and Colleagues,

It is a great honor for us to have the opportunity to introduce this Special Issue on Bariatric Surgery to *Journal of Clinical Medicine*.

Obesity is currently considered a major health problem, causing an ongoing and decades-long pandemic, which the WHO has termed the “global obesity epidemic” [1]. Although lifestyle modifications and pharmacological interventions are often exploited as first-line interventions, it is clear that bariatric surgery is the most effective strategy, especially in more severe forms of obesity [2]. In this respect, thousands of bariatric procedures (restrictive, malabsorptive, and mixed) are currently performed around the world with exponentially increasing trends and improved outcomes. During recent years, intense scientific activities in this field have been realized, allowing us to obtain more in-depth knowledge regarding the effect of bariatric procedures on obesity comorbidities, a more complete view on perioperative complications, medium-term effectiveness, and new bariatric surgery techniques, among others.

However, there are several dark spots in the field of bariatric surgery that should be better analyzed. For example, the drawbacks of one of the most performed procedures, laparoscopic sleeve gastrectomy, still need to be investigated. Several authors have underlined the worrying increase in postoperative reflux disease after the aforementioned surgery sometimes leading to cases of Barrett’s esophagus. Therefore, the literature debate is ongoing, for example, regarding the effectiveness of associating an anti-reflux plication to the conventional sleeve gastrectomy [2]. Another issue is the long-term effectiveness of the different bariatric techniques and the debated question about the weight regain of insufficient weight loss especially after restrictive procedures [3].

Notably, new malabsorptive procedures, such as the one anastomosis gastric bypass, a recent and increasingly common technique, are not free from worrying medium- and long-term sequelae such as chronic gastritis, remnant ulcers with severe bleeding, and malnutrition, which deserve deep reflection and scientific debate [4,5].

The scope of this topical collection is to provide an overview of the recent advances in the field of bariatric surgery and to provide responses to some of the open questions still present in the world of bariatric surgery. Therefore, we encourage researchers in the field of bariatric surgery to submit an original article (no animal research) or review to this topical collection.
